# Towards mental health as a human right: The key role of lived experience

**DOI:** 10.1371/journal.pmed.1004307

**Published:** 2023-10-10

**Authors:** Louise Gaynor-Brook, on behalf of the PLOS Medicine Editors

**Affiliations:** Public Library of Science, San Francisco, California, United States of America and Cambridge, United Kingdom

October 10th marks World Mental Health Day, for which the 2023 global campaign theme is ‘Mental health is a universal human right’. The World Federation for Mental Health, founded in the same year as the Universal Declaration of Human Rights was adopted by the United Nations General Assembly, urges us all — as individuals, communities, nations, and global citizens — to advocate for recognition of mental health as a fundamental human right [1].

Mental health is a core component of the World Health Organisation (WHO) Constitution, which defines health as “a state of complete physical, mental and social well-being and not merely the absence of disease or infirmity” [2]. In reality, mental health lacks the prominence it warrants in the healthcare agendas and budgets of many nations, particularly in low- and middle-income countries. Indeed, in a world where progress towards universal health coverage is reversing, with almost 2 billion people facing impoverishing levels of spending on healthcare [3], universal mental health coverage seems a distant aspiration.

It has been estimated that mental health conditions (not including substance use disorders) affect approximately 12% of the global population [4], although it is likely that many more go undiagnosed due to factors such as unavailability of mental healthcare services and reluctance to seek help due to social stigma and fear of discrimination. The most common substance use disorders are alcohol use and opioid dependence, which can have profound consequences for physical health. In 2019, opioid misuse accounted for 80% of drug-related deaths worldwide, with a 1040% increase in opioid-related deaths in the United States compared to 2013, and further increases during the COVID-19 pandemic [5]. Whilst the impact of the pandemic on mental health more generally is not clear-cut, evidence suggests that contextual factors such as mandated social isolation through repeated lockdowns and inaccessibility of routine healthcare led to an increased prevalence of mood and anxiety disorders in particular [6].

As it stands, the average government expenditure on mental health accounts for only 2.1% of healthcare spending, ranging from 1.05% to 3.8% in low- and high-income countries respectively [7]. There are signs that the importance of mental health is being recognised, with substantial increases in the proportion of countries that have devised specific health policies pertaining to mental health [7], such as in Nigeria, and recent changes to legislation in Ghana, Malaysia, Guyana and Pakistan to decriminalise suicide, removing a deterrent to individuals seeking mental healthcare when it is most needed [8]. Whilst these are undoubtedly promising developments, commitments by governments across the globe to provide the investment needed to fully implement mental health policies lag behind, despite evidence demonstrating net economic benefits of approximately three-fold returns for every $1 invested in mental healthcare [9].

Of course, mental health cannot be considered in isolation. It is well established that depression and anxiety are more prevalent among individuals with long-term physical health conditions, such as cardiovascular disease, diabetes, cancer, HIV, arthritis and multimorbidity. For some of these, evidence supports a bidirectional relationship which may be mediated at least in part by health behaviours such as healthcare avoidance, and lifestyle choices such as diet, alcohol use and physical activity. It follows that mental health conditions are associated with reduced life expectancy [10]. The social consequences of mental ill-health are also manifold, and can include withdrawal and social isolation, damage to personal relationships, risks to educational attainment and employment, and being subjected to stigmatisation and discrimination.

The Universal Declaration of Human Rights states that everyone has the right to education, to work, and to a sufficient standard of living to maintain their health and well-being, including access to food, clothing, housing and medical care. Given the interlinked nature of mental health and each of these factors, it is unquestionable that mental wellbeing should be considered a universal human right in itself. Recognition of this status must translate into steadfast commitments by governments across the globe to prioritise and invest in high-quality healthcare for the prevention and treatment of mental health conditions and, more broadly, to tackle the underlying socioeconomic factors which impact and are impacted by mental ill-health.

Recent steps towards improving equality in mental health provision at a global level include the revision of the WHO Model Lists of Essential Medicines to include additional therapeutic alternatives for mental health and behavioural conditions [11]. Medicines included on this list are intended to be affordable and accessible across all health systems, thereby improving the availability of medications that are more effective and/or more acceptable to individual patients. However, the optimal approach to treating mental health conditions extends well beyond pharmacological methods and includes concurrent resource-intensive psychotherapies, especially for mood, anxiety and substance-use disorders. The onset of approximately 60% of mental health conditions before the age of 25 years [12] also necessitates an urgent rethink around efforts towards prevention, especially in adolescents and young adults. With limited resources to improve mental health, particularly in the wake of COVID-19, it is essential that healthcare budgets are spent wisely for maximal benefit.

People with lived experience of mental health conditions can offer invaluable holistic perspectives on the accessibility, acceptability and effectiveness of mental health services, unmet needs and priorities, approaches to improving continued engagement with mental healthcare, and insights into experiences of stigmatisation and discrimination, among many others. In addition to informing how mental health services could be refined, the potential for people with lived experience to actively contribute to service delivery alongside mental healthcare professionals, such as through providing peer-led psychotherapies, is increasingly being explored [13,14]. Strategies and policies relating to mental healthcare and priorities within research agendas should be developed alongside people with lived experience to ensure that the insights and values of those affected are fully taken into account. Through recognising the importance of the principle “nothing about us, without us”, there is potential to inform redesign of mental healthcare services to more closely meet the needs of those using them, to further efforts to tackle stigma which deters many from seeking help at all, to promote preventive approaches and more effectively signpost to mental health services where needed, to improve continued engagement with treatment and, ultimately, to extend the reach and benefit of mental healthcare that is so urgently needed.

*PLOS Mental Health is a new journal focused on advancing mental health research*, *treatment and care by putting lived experience of individuals and communities at the forefront of their mission*. *PLOS Mental Health opens for submissions later this year*: *please see* *https*:*//plos*.*org/mental-health-research-journal* *for more information*.
